# 566. Impact of a Culturally Sensitive Multilingual Community Outreach Model on COVID-19 Vaccinations at an Urban Safety-net Community Hospital

**DOI:** 10.1093/ofid/ofab466.764

**Published:** 2021-12-04

**Authors:** Alfredo J Mena Lora, Stephanie L Echeverria, Ella Li, Miguel Morales, Rita Esquiliano, Genessa Schultz, James Sifuentes, Sherrie Spencer, Eden Takhsh, Romeen Lavani

**Affiliations:** 1 University of Illinois at Chicago, Chicago, Illinois; 2 Saint Anthony Hospital, Chicago, Illinois

## Abstract

**Background:**

The United States (US) is one of the most affected countries by the COVID-19 pandemic. A disproportionate burden of COVID-19 deaths is seen in Black, Asian, and Latinx groups. COVID-19 vaccines are the primary mitigation strategy to reduce morbidity and mortality. However, vaccine hesitancy is high in these communities due to factors such as low health literacy, language barriers, and other health inequities. Our objective was to implement a culturally sensitive, multi-lingual, community outreach model to promote vaccine education and facilitate vaccine administration.

**Methods:**

Community healthcare workers or “promotoras” were deployed to high traffic areas such as supermarkets, laundromats, churches, and commercial hubs from February-May 2021. The promotoras provided culturally sensitive vaccine counseling to individuals in their preferred language and facilitated vaccine appointments at our hospital. Our data was compared with publicly available data from other facilities organized by ZIP codes defined by the Department of Public Health as low, medium, or high-vulnerability to COVID-19.

**Results:**

A total of 109 outreach workers were hired, of which 67% (73) were Latinx, 27% (29) Black and 6% (7) Asian. Overall, 8,806 individual encounters led to 6,149 scheduled appointments and 3,192 completed first doses (Figure 1). A total of 14,636 individuals were vaccinated. Average age was 45.5 (range 12-98). Preferred language was 54% Spanish, 38% English, and 8% Chinese. Ethnicity was mostly Hispanic (66%) with race mostly white (54%) (Figure 2). High and medium-risk ZIP codes represented 69.4% of vaccinations at our facility (Figure 3).

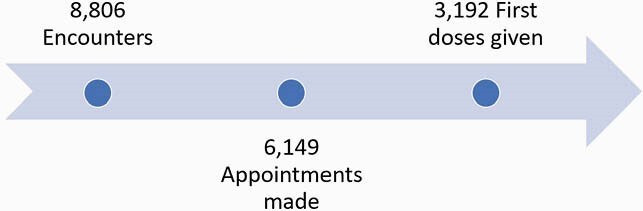

Figure 1. Education encounters and appointments made by community outreach workers and associated vaccinations.

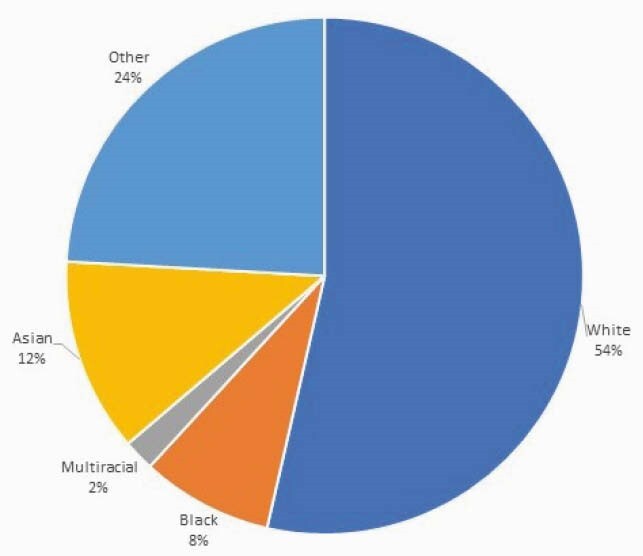

Figure 2. Racial distribution of vaccinated individuals at our facility

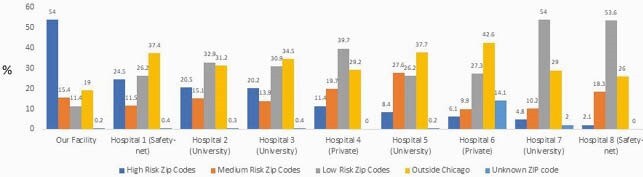

Figure 3. Comparative vaccinations by zip codes from hospitals in our area.

**Conclusion:**

We successfully implemented a culturally sensitive community outreach model which resulted in higher vaccination rates from at risk ZIP codes when compared to other hospitals. Promotoras encouraged vaccination in native languages, thereby increasing vaccine awareness and appointment faciliation. Barriers to vaccine access remain in these vulnerable communities. This model educated the community via its own members and may help reduce barriers, increase vaccine awareness and vaccination rates.

**Disclosures:**

**All Authors**: No reported disclosures

